# Hybrid Perovskite Solar Cells: A Disruptive Technology for Hydrogen Production through Photocatalytic Water Splitting

**DOI:** 10.1002/open.202500181

**Published:** 2025-05-24

**Authors:** S. Akin Olaleru, Nithyadharseni Palaniyandy, Bhekie B. Mamba, Bonex W. Mwakikunga

**Affiliations:** ^1^ Institute for Nanotechnology and Water Sustainability (iNanoWS) College of Science, Engineering and Technology University of South Africa Florida 1709 South Africa; ^2^ Physics Department Yaba College of Technology P.M.B 2011 Lagos 100001 Nigeria; ^3^ Institute for Catalysis and Energy and Solutions (ICES) College of Science, Engineering and Technology University of South Africa Florida 1709 South Africa; ^4^ DST/CSIR‐National Centre for Nano‐Structured Materials P.O. Box 395 Pretoria 0001 South Africa; ^5^ Department of Physics Arcadia Campus Tshwane University of Technology Pretoria 0001 South Africa

**Keywords:** disruptive technology, hydrogen production, low‐carbon transition, perovskite solar cell, water splitting

## Abstract

Perovskite solar cells (PSCs) have recently emerged as a viable technology for photovoltaic applications, offering high efficiency and cost‐effective manufacturing. Beyond generating electricity, PSCs can also facilitate hydrogen production through water splitting. This article provides a comprehensive review of current research on PSCs for hydrogen production, highlighting their potential as a transformative technology in this field. The challenges and opportunities associated with using PSCs for hydrogen production are discussed, including their stability and efficiency under various operating conditions. The impact of device design, system integration, and materials engineering on PSC performance for hydrogen production is also examined. Furthermore, an overview of hydrogen demand is provided and how PSCs can be integrated with other renewable energy sources to contribute to a sustainable energy future through green hydrogen production is explored. The analysis suggests that hydrogen production using PSCs has the potential to become a groundbreaking technology, significantly impacting the energy sector and the transition to low‐carbon energy.

## Graphical abstract

This study demonstrates the potential of hybrid perovskite solar cells for photocatalytic water splitting and sustainable hydrogen production. By combining bandgap engineering and heterojunction design, enhanced efficiency and clean energy generation are achieved, advancing next‐generation solar‐driven hydrogen technologies.

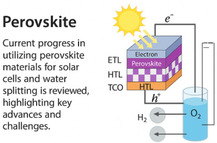

## 1 Introduction

One of the most pressing issues facing humanity in the 21st century is addressing the global energy crisis. The United Nations Secretary‐General emphasizes the interconnectedness of energy with globalization, social justice, and environmental sustainability.^[^ ^1^, ^2^ ^]^ The current energy infrastructure poses two significant challenges: meeting increasing energy demand due to population growth and transitioning to sustainable energy sources. The global population, currently 7.8 billion, is projected to reach ≈9 billion by 2040,^[^ ^3^ ^]^ further straining energy resources. Notably, about 1 billion people lack access to electricity,^[^ ^2^, ^4^ ^]^ while 40% of the global population relies on solid fuels and polluting technologies for cooking, lighting, and heating.^[^ ^2^, ^5^, ^6^, ^7^ ^]^

Fossil fuels, which dominate the current energy mix, are finite resources consumed at a rate far exceeding their natural replenishment. The combustion of fossil fuels contributes to global warming and climate change through the emission of carbon dioxide (CO_2_) and other greenhouse gases. To mitigate climate change, greenhouse gas emissions must be reduced by 45% by 2030 and reach net zero by 2050 to limit global warming to 1.5 °C above preindustrial levels.^[^ ^1^, ^8^, ^9^ ^]^ This necessitates a rapid transition to clean energy sources and a phase‐down of fossil fuels.^[^ ^1^, ^10^ ^]^

**Figure 1:**
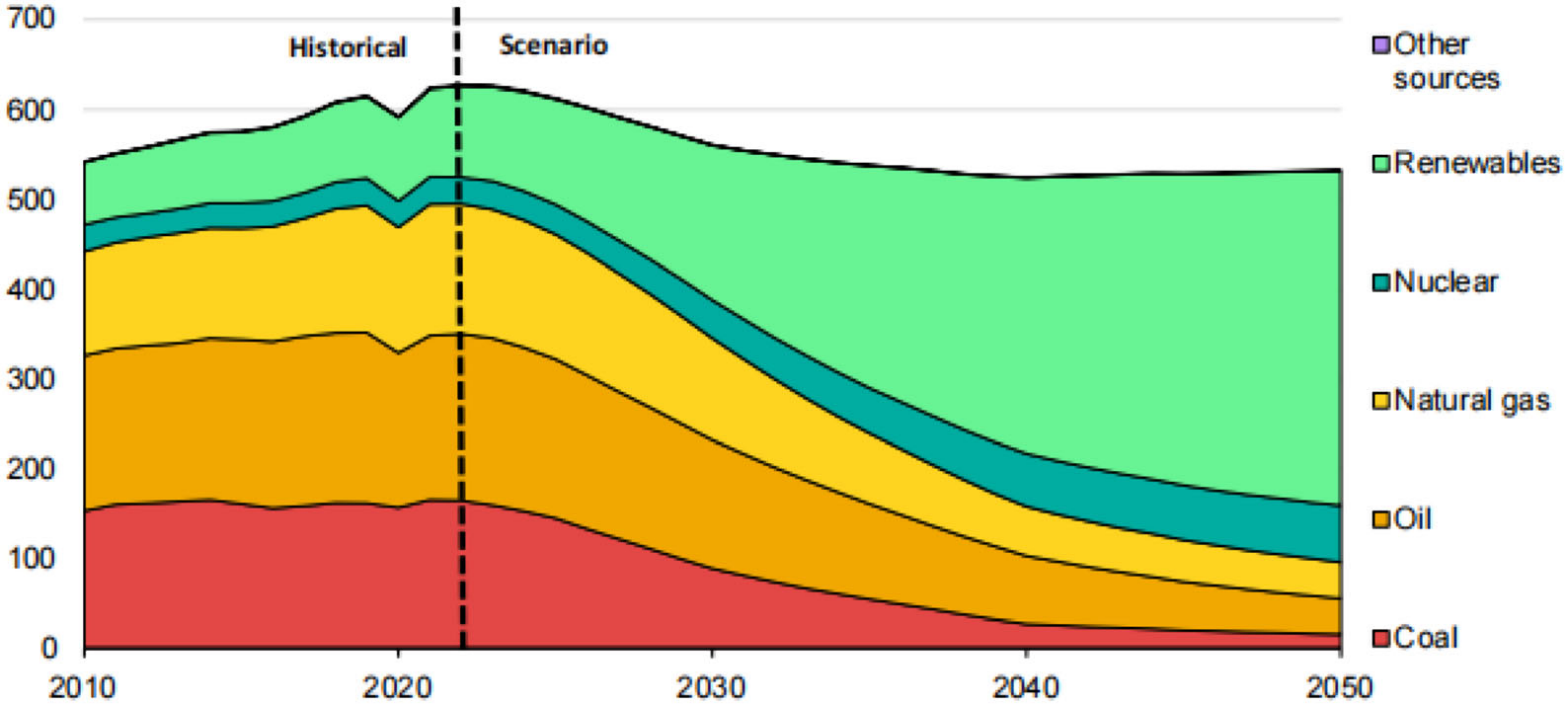
A large portion of fossil fuel use is displaced by nuclear and renewable energy in the NZE Scenario, with fossil fuel use plummeting from almost 80% in 2021 to less than 20% in 2050.^[^ ^12^ ^]^

A recent report by the World Meteorological Organization (WMO), a collaborative effort involving 26 organizations, highlights energy as a crucial factor in achieving global agreements on sustainable development and climate change, as well as preserving planetary health.^[^ ^11^ ^]^ Energy is indeed a common thread. To meet global goals, electricity generation from clean energy sources must double by 2030; otherwise, energy security and renewable energy targets are at risk.^[^ ^1^, ^10^, ^11^ ^]^ The widespread reliance on fossil fuels has led to energy shortages and severe pollution problems, prompting a global search for viable alternatives to create a green and renewable energy economy. Renewable energy sources, such as solar energy, are being extensively researched to address these challenges^[^ ^12^ ^]^ as seen in **Figure** **1**.

Solar‐powered fuels offer a promising solution to meet global energy demands by converting and storing intermittent solar energy. Solar water splitting is a common method for producing hydrogen, a clean energy carrier with high energy density and favorable environmental properties.^[^ ^13^ ^]^ Hydrogen has gained recognition as a potential replacement for conventional energy sources due to its clean energy conversion process. However, current hydrogen production methods, such as thermochemical production, often rely on fossil fuels or cause environmental harm.^[^ ^14^, ^15^ ^]^ To increase hydrogen use sustainably, production methods based on renewable energy sources must be developed further. Environmentally safe hydrogen production is a major challenge hindering the development of hydrogen‐based technologies.^[^ ^15^ ^]^

A major challenge in solar hydrogen production is identifying photo‐absorber materials with superior optoelectronic properties, which limits the scalability of this technology.^[^ ^16^, ^17^ ^]^ Despite research efforts, material instability, low efficiency, and high manufacturing costs remain significant obstacles to widespread adoption.^[^ ^13^, ^18^, ^19^ ^]^ Developing new materials for efficient solar hydrogen production is crucial, and perovskite materials show promise. This review hypothesizes that optoelectronic properties beneficial for photovoltaics (PV) are also relevant for photocatalysis. However, few studies have explored hybrid perovskites for photocatalysis^[^ ^13^, ^20^, ^21^ ^]^ likely due to their instability in certain media. Solar‐driven water splitting offers a cleaner approach to hydrogen production, emitting no toxic gases and scalable potential.

**Figure 2:**
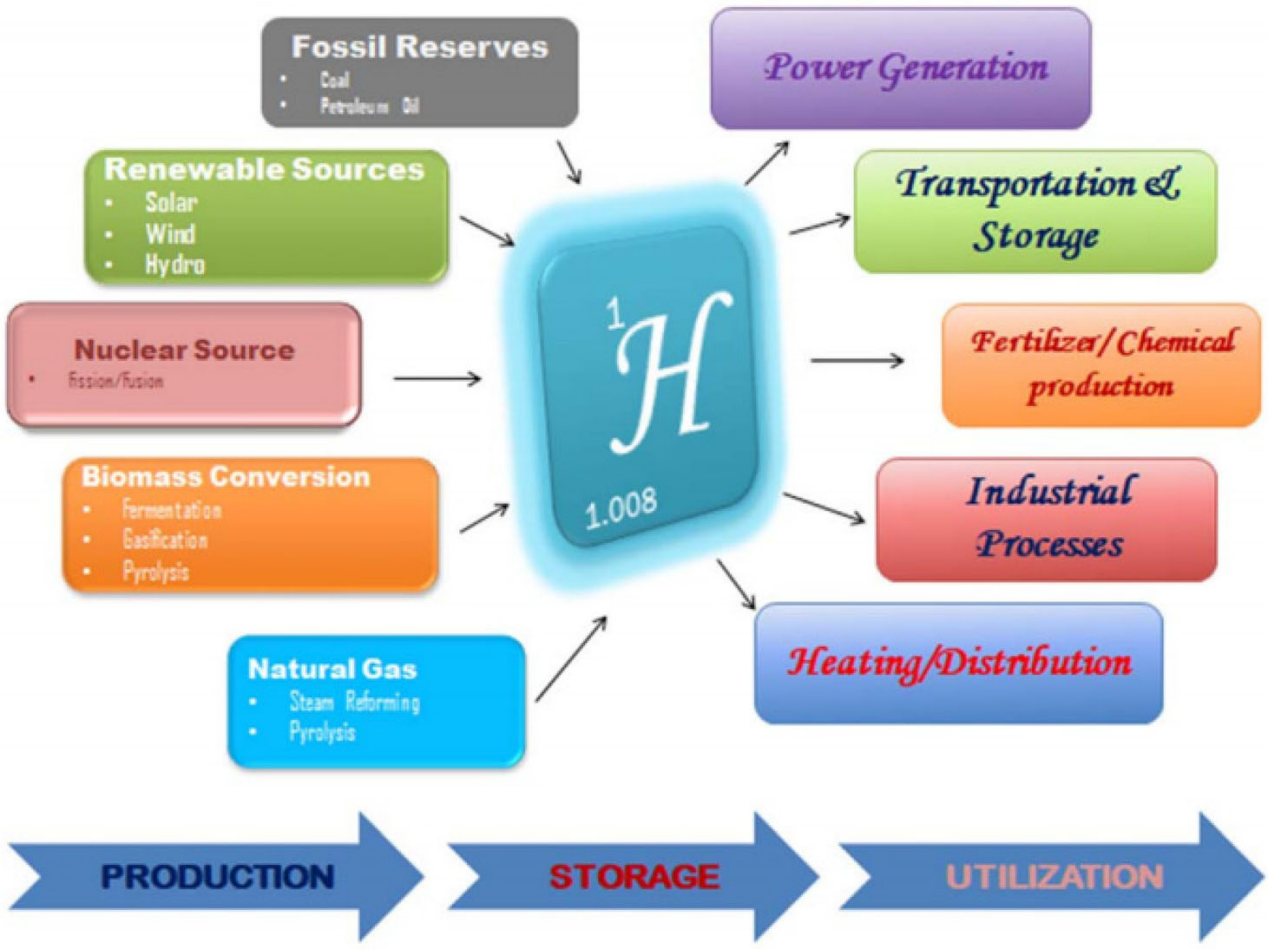
Several available hydrogen‐generating methods. Reproduced with permission.^[^ ^23^ ^]^

Several strategies can be employed for water splitting, including photocatalysis, electrocatalysis, photoelectrochemistry, thermochemistry, and electrochemistry with PV assistance.^[^ ^13^, ^22^ ^]^ Other methods rely on thermal energy sources, as illustrated in **Figure** **2**.^[^ ^23^ ^]^ This review focuses on solar‐driven water splitting, a clean and efficient method for hydrogen production. Our objective is to provide insight into water splitting using perovskite solar cells for hydrogen generation and to evaluate the current state of sustainable hydrogen production approaches. Furthermore, we investigate the potential of PSCs as a transformative technology for hydrogen production.

This study aims to advance the understanding of water splitting using perovskite solar cells for hydrogen generation by reviewing current developments in sustainable hydrogen production approaches. We investigate the potential of PSCs as a transformative technology for hydrogen production. Renewable energy‐based hydrogen production is crucial for meeting the growing demand for clean energy. PSCs offer a viable alternative to conventional silicon solar cells due to their low cost, high efficiency, and tunable optical and electrical properties. This analysis examines recent advancements in PSC technology and evaluates its potential for hydrogen production via water splitting, highlighting the stability, scalability, and cost‐effectiveness of PSC‐based hydrogen production.

## 2 Materials Profile for Hybrid Perovskite: Structure and Properties

**Figure 3:**
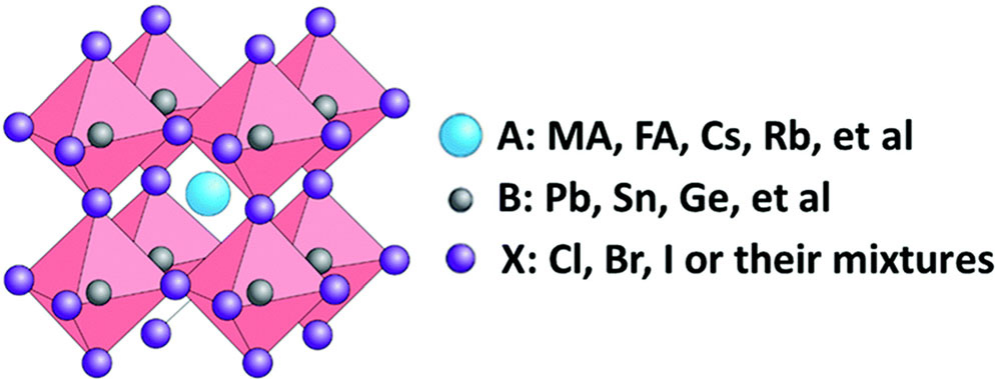
A unit cell of ABX_3_ perovskite structure.^[^ ^135^ ^]^

Hybrid perovskites are materials that combine organic and inorganic elements, forming a crystal structure represented by the general formula ABX_3_, where A is an organic molecule, B is a metal ion, and X is a halide. These materials typically consist of compounds such as methylammonium lead iodide (CH_3_NH_3_PbI_3_, MAPbI_3_) or formamidinium lead iodide (FAPbI_3_). The inorganic and metallic components are bound together by strong covalent bonds in a lattice‐like structure conferring unique physical and chemical properties. Hybrid perovskites have garnered significant attention for their potential applications in PV, light‐emitting diodes, and optoelectronics. **Figure** **3** depicts the perovskite crystal structure. A material suitable for solar hydrogen production should possess a narrow bandgap, long carrier diffusion length, and excellent surface reaction kinetics in solar energy conversion, which involves harvesting solar light, charge separation, and surface catalytic reaction.^[^ ^24^, ^25^, ^26^ ^]^

Hybrid perovskites have been extensively researched due to their exceptional optoelectronic properties, including high light absorption coefficients, tunable bandgaps, and high carrier extraction efficiency, with reported efficiencies exceeding 26%.^[^ ^27^ ^]^ Lead halide perovskites, in particular, exhibit promising optoelectronic characteristics, such as exceptionally long carrier diffusion lengths (475 nm, the charge carrier diffusion length of MAPbI_3_ is an order of magnitude greater than that of GaAs).^[^ ^28^, ^29^ ^]^ Certain oxide perovskites can be employed in photocatalytic processes, but their limited use is due to wide bandgaps and poor optoelectronic properties, particularly low electron/hole mobility.^[^ ^13^ ^]^ Hybrid perovskites are promising materials for solar water splitting due to the following reasons: i) Hybrid perovskites have the ability to capture a maximum amount of light in the form of tiny particles or thin films thanks to their high light absorption coefficient.^[^ ^30^, ^31^ ^]^ ii) Hybrid perovskites are desirable from a thermodynamic point of view for solar‐hydrogen reaction conditions, as they are able to precisely tune bandgaps and band edges by changing the chemical composition (e.g., by simply exchanging the cations in the crystal lattice or by developing a wide range of oxygen or halogen non‐stoichiometries). In addition, hybrid perovskites can utilize more visible sunlight by reducing the bandgaps through bandgap engineering.^[^ ^13^ ^]^ iii) Better charge separation in the bulk is enabled by less recombination due to the long carrier diffusion length given by the special properties of hybrid perovskites.^[^ ^32^, ^33^, ^34^ ^]^ In addition, the ferroelectric and piezoelectric properties of some hybrid perovskites can be further improved by changing the charge separation method.^[^ ^35^, ^36^ ^]^ iv) For the commercial utilization of hybrid perovskites for solar hydrogen generation, the cost of fabricating perovskite‐based devices with the solution method is much lower than the cost of fabricating standard solar cells.^[^ ^37^, ^38^ ^]^ Hybrid perovskites are essential for solar water splitting.

Hybrid perovskite solar cells typically consist of multiple functional layers, including a transparent conductive oxide (TCO) electrode, an electron transport layer (ETL) such as titanium dioxide (TiO_2_) or tin dioxide (SnO_2_), a perovskite light‐absorbing layer (e.g., MAPbI_3_ or FAPbI_3_), a hole transport layer (HTL) like spiro‐OMeTAD, and a metal back contact. Their exceptional optoelectronic properties, including high absorption coefficients, tunable bandgaps, and long charge‐carrier diffusion lengths, enable power conversion efficiencies (PCEs) exceeding 26% in laboratory settings, comparable to traditional silicon‐based solar cells. The performance of PSCs has advanced rapidly, positioning them as a leading candidate for next‐generation PV. Evaluating PSC performance involves assessing efficiency, stability, scalability, and material adaptability, as outlined in **Table** **1**.^[^ ^27^, ^39^ ^]^ **Table** **2** presents a comparative analysis of light absorber materials for solar cells, examining the properties, advantages, efficiency, and challenges of perovskites, silicon, organic, and dye‐sensitized systems.^[^ ^27^, ^39^, ^40^ ^]^

**Table 1:** PSC performance can be summarized using key metrics.

Parameter	Current status
Efficiency (single junction)	>26%
Efficiency (tandem)	>30%
Bandgap	Tunable (1.5–2.3 eV)
Carrier lifetime	Up to microseconds
Carrier diffusion length	Several micrometers
Stability	Improving, but still a major challenge
Fabrication cost	Low (solution processing compatible)

**Table 2:** Comparison of light absorber materials in solar cells.

Material	Max PCE [%]	Key advantages	Challenges
Perovskites (e.g., MAPbI_3_, FAPbBr_3_)	≈26%	High PCE, low‐cost fabrication, tunable bandgap	Stability, lead toxicity, moisture sensitivity
Silicon (Si)	>26%	Mature tech, long‐term stability, widely available	High purification and fabrication costs
Organic (OPVs)	≈18%	Lightweight, flexible, printable	Lower operational stability
Quantum dots (QDs)	≈16%	Tunable bandgap, potential MEG	Toxicity (Pb, Cd), stability
Dye‐sensitized (DSSCs)	≈14%	Transparent, good indoor/low‐light performance	Liquid electrolyte instability
CZTS/CZTSe	≈12%	Earth‐abundant, nontoxic	Interface defects, low Voc
Antimony selenide (Sb_2_Se_3_)	≈10%	Simple structure, stable	Limited carrier mobility and diffusion
TMDCs (MoS_2_, WS_2_, etc.)	<5%	2D structure, ultrathin, direct bandgap in monolayer	Low efficiency, processing challenges
Plasmonic nanomaterials	Varies	Light trapping, boosts absorption in hybrids	Cost of noble metals, integration

### 2.1 Materials Profile for Hybrid Perovskite: Problems

Stability and toxicity are two decisive factors for water splitting with perovskite solar cells for commercial applications.

#### 2.1.1 Stability

Hybrid perovskites are promising materials for solar‐to‐fuel conversion due to their superior optoelectronic properties. However, their instability in photocatalytic environments hinders commercialization. Recent reviews have extensively discussed the decomposition mechanisms of hybrid perovskite‐based solar cells.^[^ ^41^, ^42^ ^]^ Notably, the working environment for photocatalysis differs significantly from PV applications. Two major constraints limit the use of perovskite photocatalysts: 1) instability in polar solvents and 2) surface reactivity with chemical species in solution.^[^ ^41^, ^42^ ^]^ Perovskite stability concerns can be categorized into intrinsic stability (structural, electronic, and thermodynamic phase stability) and extrinsic stability (interactions with water, degradation pathways, thermochemical stability, light‐induced effects, light‐induced ion redistribution, light stability, photochemical degradation, and oxidation of transport layers and perovskites).^[^ ^43^ ^]^ The softness of the perovskite material arises from weak ionic bonds, such as the Pb—I bond with a bonding energy of 142 kJ mol^−1^,^[^ ^44^ ^]^ as well as secondary interactions like van der Waals forces and hydrogen bonding.^[^ ^45^ ^]^

Perovskite solar cells are inherently sensitive to moisture, heat, light, electric fields, and oxygen, leading to degradation.^[^ ^46^ ^]^ Niu et al. demonstrated that iodide perovskites are particularly susceptible to moisture, with a negative standard Gibbs free energy for moisture degradation.^[^ ^47^ ^]^ Hybrid perovskites’ instability stems from weak van der Waals interactions, ion migration, photodegradation, and phase segregation. Additionally, weak hydrogen bonds contribute to poor intrinsic stability and a soft ion lattice. To improve stability and photocatalytic performance, various approaches have been explored, including encapsulation in protective layers, passivation, and additive incorporation. Encapsulation is a promising method for enhancing both stability and photocatalytic efficacy. Composition engineering, which involves substituting anions or cations in the ABX_3_ crystal structure, has also proven effective in boosting stability and performance.^[^ ^48^, ^49^ ^]^ To enhance the device's optoelectronic properties, composition engineering involves partial or complete substitution of suitable materials for the anion or cation in the perovskite crystal structure, ABX_3_.

#### 2.1.2 Toxicity

The toxicity of lead halide perovskites is a significant concern for commercialization. Researchers are actively exploring lead‐free alternatives, with promising results.^[^ ^50^ ^]^ Currently, high‐performance PSCs are based on lead halide perovskites, which pose potential toxicity risks.^[^ ^50^ ^]^ Potential solutions involve substituting lead with elements like Sn, Ge, Bi, or Sb.^[^ ^50^ ^]^ Among these alternatives, Sn‐based PSCs have shown the most promise, although their performance lags behind Pb‐based PSCs due to stability and energy level mismatch issues.^[^ ^49^, ^51^ ^]^ Further research is needed to improve the efficiency of lead‐free PSCs.^[^ ^52^ ^]^ To mitigate toxicity concerns, preventive measures have been recommended, including proper end‐of‐life disposal (e.g., recycling) and adequate device encapsulation.^[^ ^53^ ^]^ Overall, perovskite research is still in its developmental stage, requiring significant advancements in material performance and stability. Nevertheless, prospects for perovskite materials are promising, driven by ongoing progress in materials science and technology.

#### 2.1.3 Scaling up and Reproducibility Challenges

Scaling up perovskite solar cells to meet commercial demands poses significant challenges. Although the materials are relatively inexpensive, the fabrication process requires precise control over environmental conditions. Conventional spin‐coating methods are limited to small‐area devices, necessitating the development of reliable and reproducible manufacturing techniques for large‐area modules. Key challenges include the following: i) developing scalable deposition techniques to uniformly coat all layers (perovskite absorber, transport layers, and electrodes) over large areas, matching the uniformity of spin‐coating for small‐area cells; ii) modifying precursor solution chemistry to maintain control over film formation during scale‐up processes; and iii) designing module architectures that differ from those used for small‐area cells.^[^ ^54^ ^]^ Despite these challenges, scalable techniques like blade coating^[^ ^55^ ^]^ have demonstrated performance comparable to spin‐coating methods, highlighting the potential for scaling up perovskite solar cells. Techniques for fabricating large‐area PSCs include slot‐die coating, inkjet printing, blade coating, screen printing, spray coating, vapor‐phase deposition, and electrodeposition.^[^ ^54^, ^55^, ^56^, ^57^, ^58^, ^59^, ^60^, ^61^, ^62^, ^63^, ^64^, ^65^, ^66^, ^67^, ^68^, ^69^, ^70^ ^]^ Some methods are compatible with roll‐to‐roll (R2R) printing, while others require screen‐to‐screen (S2S) processing.^[^ ^54^ ^]^

The performance difference between small‐ and large‐area perovskite solar cells can be attributed to variations in film shape and quality resulting from spin‐coating versus scale‐up techniques like blade coating or slot‐die coating. However, recent advancements in ink formulation and optimized annealing/drying conditions have narrowed the performance gap between small‐area cells and large‐area modules.^[^ ^54^ ^]^ As reported by Jena et al.^[^ ^54^ ^]^ notable successes have been achieved in large‐area perovskite solar modules, demonstrating progress in scaling up these technologies.

## 3 Hydrogen H_2_: Overview

Hydrogen is a fundamental element that readily forms compounds with other elements, making it ubiquitous in water, hydrocarbons, and biomass. As an energy carrier rather than a source, hydrogen's popularity is growing globally as a potential fuel for the future.^[^ ^71^, ^72^ ^]^ Governments are investing in hydrogen production via Carbon Capture Usage and Storage (CCUS) and water electrolysis to reduce greenhouse gas emissions.^[^ ^73^ ^]^ The global energy crisis, exacerbated by the Russia‐Ukraine conflict, has accelerated interest in low‐emission hydrogen for decarbonization and energy security. While still in development, low‐emission hydrogen holds promise as a clean industrial feedstock and energy source. Effective tracking of its development is crucial to determine its potential contribution to the clean energy transition.

As the world transitions to decarbonized energy systems, hydrogen will play a critical role as a clean energy carrier. Its use in fuel cells offers a significant advantage, producing only water as a byproduct, making it ideal for urban applications such as buses and cogeneration units in buildings. Currently, hydrogen is primarily used in refineries, ammonia production, and steel manufacturing, as well as chemical feedstock. This report aims to support decision‐makers in planning and securing funding for low‐emission hydrogen projects, facilitating its adoption.

### 3.1 Hydrogen Demand

**Figure 4:**
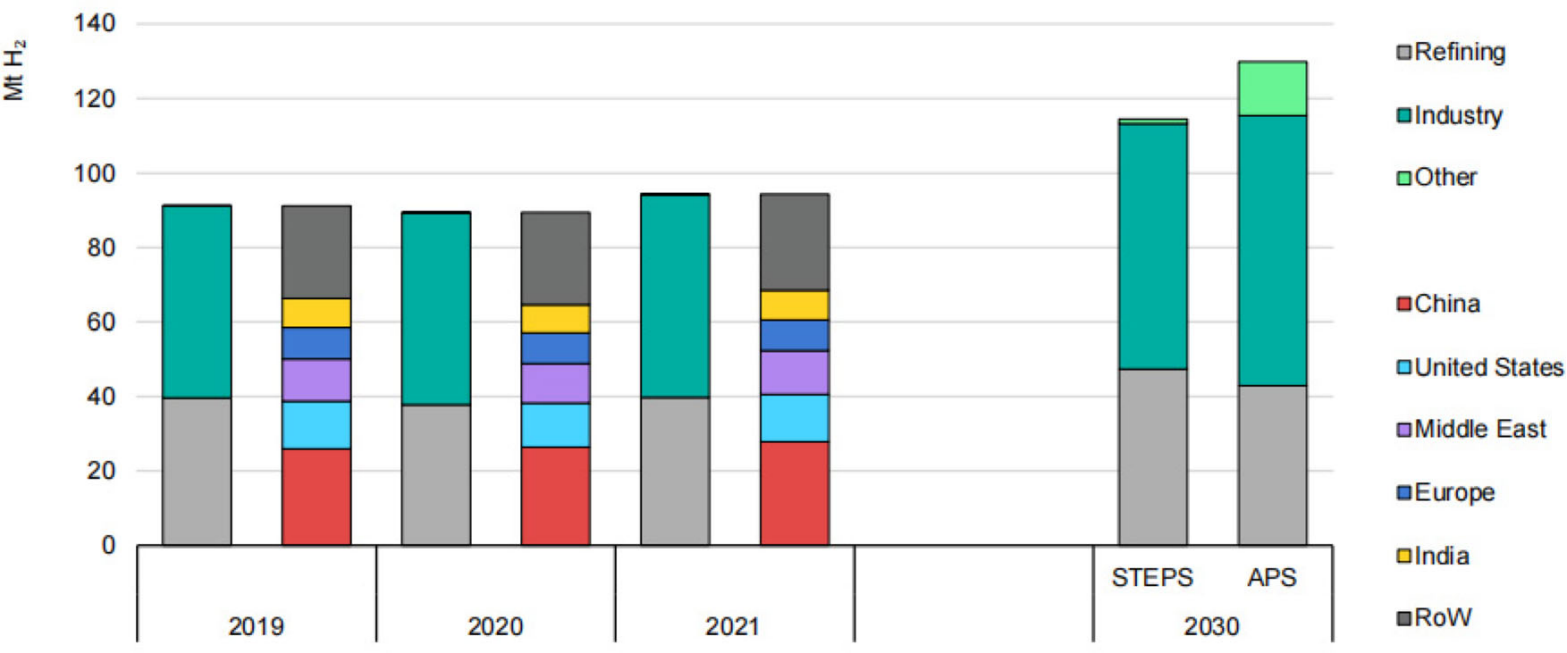
According to the Stated Policies and Announced Pledges scenarios, hydrogen consumption varies by industry and location from 2019 to 2030.

Global hydrogen demand has experienced a significant increase in recent years, driven by the growing utilization of gas for energy applications. According to the International Energy Agency (IEA), global hydrogen demand reached 91 million tons of oil equivalent (Mtoe) in 2019 and 94 million tons (Mt) in 2021, representing a 5% increase from the previous year.^[^ ^73^ ^]^ Notably, demand is projected to escalate to 115 Mtoe by 2030. The primary applications of hydrogen include ammonia production, methanol synthesis, and petroleum refining, with the chemical and refining industries accounting for ≈78% of global demand. The transportation and industrial sectors are anticipated to exhibit the most rapid growth, with average annual growth rates of 2.5% and 2.3%, respectively. Geographically, the Asia–Pacific region dominates the global hydrogen market, accounting for 59% of total demand, followed by Europe (25%), North America (12%), and the Middle East (4%).^[^ ^73^ ^]^ The increasing adoption of hydrogen as a clean energy source is expected to be driven by advancements in fuel cell technology (**Figure** **4**). Global hydrogen consumption increased by 5% in 2021, reflecting the rebound in economic activity in traditional applications following pandemic‐related constraints^[^ ^73^ ^]^

### 3.2 Achieving Hydrogen's Potential

The global demand for hydrogen continues to escalate; however, the current supply is predominantly comprised of nonlow‐carbon hydrogen, with blue hydrogen being the sole low‐carbon variant. Substantial advancements are requisite for hydrogen to make a meaningful contribution to the energy transition. A comprehensive review of the literature^[^ ^74^ ^]^ highlights four critical challenges that must be addressed to actualize the potential of hydrogen: scaling competitive supply chains, stimulating domestic demand, advancing hydrogen transportation technologies, and facilitating cross‐value chain business collaborations. 1) Scaling competitive hydrogen supply: To achieve this objective, countries and organizations (HRCs) must deploy both blue and green hydrogen. In the short to medium term, blue hydrogen will play a crucial role due to its current economic feasibility, while green hydrogen will become increasingly important in the long term as costs decline. Significant reductions in blue hydrogen production costs are anticipated by 2030, driven by technological advancements in carbon capture, utilization, and storage (CCUS) and access to affordable natural gas or hydrocarbons. Complementary renewable energy sources, such as solar power, and declining capital costs for electrolyzers will also contribute to cost reductions. According to the Hydrogen Council, the cost of hydrogen for end‐users is projected to decrease by 60% between 2020 and 2030.^[^ ^75^ ^]^ Furthermore, the International Renewable Energy Agency (IRENA) provides a basis for anticipating continued cost reductions through 2030 and 2050.^[^ ^76^ ^]^ 2) Stimulating local demand: The development of a local hydrogen market is crucial to establishing a robust hydrogen ecosystem, complementing export infrastructure. Governments can facilitate the emergence of local demand sectors by implementing regulatory frameworks that promote decarbonization and clean air initiatives. According to McKinsey,^[^ ^73^ ^]^ by 2030, clean hydrogen‐based steel and ammonia production could become competitive with conventional methods at a carbon price of $50–100 per ton, contingent upon regional conditions. 3) Developing transportation technology: Hydrogen transportation poses significant technical and economic challenges, as it requires liquefaction or conversion to ammonia. Liquefying hydrogen is particularly complex, necessitating cooling to −252 °C, the lowest boiling point of all elements, which is both costly and technically demanding. According to a McKinsey report,^[^ ^74^ ^]^ transporting hydrogen in the form of ammonia from the Middle East to Europe and subsequent reconversion to hydrogen could increase costs by $2.50–3.00 per kilogram by 2030. This is substantial, considering the projected production cost of green hydrogen in the region may be under $2.00 per kilogram. 4) Facilitating cross‐value chain collaboration: The development of the clean hydrogen industry necessitates collaboration among stakeholders across disparate value chains, end‐users, and nations. Strategic partnerships and long‐term off‐take agreements between hydrogen producers and consumers, such as steel and green fertilizer manufacturers, can effectively mitigate investment risks and foster project viability. Furthermore, partnerships that guarantee minimum offtake can incentivize investments in infrastructure and equipment. Concurrently, diplomatic relationships between governments can facilitate the establishment of international hydrogen trade frameworks, stimulate demand in target regions, and secure supply agreements.^[^ ^74^ ^]^

### 3.3 Hydrogen Production: Renewable Routes

The abundance of hydrogen in the universe contrasts with its scarcity as a free element on Earth, where it is predominantly found in molecular forms, such as water (H_2_O) and hydrocarbons, notably methane (CH_4_). To harness hydrogen, these molecules must undergo separation, enabling the recovery of free hydrogen through recombination with oxygen to form water.^[^ ^77^ ^]^ Hydrogen production can be achieved through various methodologies, utilizing both renewable and nonrenewable energy sources, as well as fossil and renewable feedstocks. To attain carbon neutrality, it is imperative to employ renewable energy sources in both the production processes and the feedstocks themselves. This section provides an overview of the primary processes employed for hydrogen production, with a particular emphasis on those that leverage renewable energy sources. Solar cells, owing to their simplicity, compactness, and relative affordability, constitute a prominent renewable energy source, particularly for household applications. Notably, perovskite solar cells have garnered significant attention due to their exceptional optoelectronic properties, which enable the generation of green power and the splitting of water for hydrogen production. Perovskite solar cells demonstrate exceptional efficiency in harnessing solar energy and converting it into electricity, thereby rendering them an attractive option for powering hydrogen production via electrolysis. Their lightweight architecture, facile manufacturability, and relatively low production costs compared to other PV technologies further enhance their appeal. Moreover, perovskite solar cells exhibit considerable potential for achieving enhanced efficiencies, thereby augmenting their suitability for driving electrolysis and extracting hydrogen from water. A plethora of research endeavors have investigated the application of hybrid perovskites in solar‐driven hydrogen production systems, encompassing particulate photocatalysis, photoelectrocatalysis, and PV electrocatalysis.^[^ ^22^, ^78^, ^79^, ^80^ ^]^ Various hybrid perovskite materials, including inorganic and organic–inorganic hybrids, have been scrutinized for their potential in facilitating solar hydrogen production.^[^ ^13^ ^]^ Prior to delving into the specifics of hydrogen generation via perovskite solar cells, it is pertinent to provide a concise overview of the color‐coded classification of hydrogen production, which denotes the energy source and production methodology employed.^[^ ^21^ ^]^ This classification encompasses the following: Gray hydrogen: produced via steam reforming of natural gas, with CO_2_ released into the atmosphere. This is indeed one of the most common forms of hydrogen production. Blue hydrogen: produced via steam reforming of natural gas, with carbon dioxide capture and storage. Green hydrogen: generated through electrolysis of water using renewable energy sources, such as wind, solar, or hydroelectric power. Pink hydrogen: produced through water electrolysis utilizing nuclear power. Black/brown hydrogen: derived from coal gasification, resulting in substantial CO_2_ emissions. Brown hydrogen: some sources might use this term to refer to hydrogen produced from coal or other fossil fuels with high carbon intensity, often associated with significant CO_2_ emissions. Turquoise hydrogen: produced from biomass resources, such as wood or agricultural waste, with carbon dioxide capture.

## 4 Similarities between Photovoltaic cells and Photocatalysis

Photocatalysis and PV share common essential steps, including light absorption, charge separation, and charge migration. However, the primary distinction between the two lies in their operational mechanisms. In photocatalysis, substrates undergo chemical oxidation and/or reduction, whereas in PV, efficient electron transfer occurs between particles and electrolytes. Given the transformative impact of hybrid perovskites on the field of PV, it is reasonable to hypothesize that their favorable optoelectronic properties may also confer high photocatalytic activity.^[^ ^81^ ^]^

## 5 Mechanisms of Water Splitting

Water splitting is a crucial chemical reaction wherein water molecules are cleaved into hydrogen and oxygen atoms. This process is pivotal in photosynthesis, enabling plants to harness solar energy. Water splitting is also a necessary step in the production of hydrogen fuel. Two essential components are required: a catalyst and an energy source. The catalyst facilitates the reaction by providing a surface for electron transfer from water molecules to oxygen and hydrogen atoms. Common catalysts include metals (e.g., nickel, cobalt, and iron) and transition metal oxides (e.g., titanium dioxide).^[^ ^41^ ^]^ The energy source provides the activation energy required for the reaction, which can be supplied by electricity, light, or heat. In photosynthesis, light energy is absorbed by chlorophyll molecules, exciting electrons and driving water splitting. This reaction is vital for producing oxygen and hydrogen, essential for life on Earth, and for generating hydrogen fuel, a potential replacement for fossil fuels.

The splitting of water yields hydrogen and oxygen molecules through an endothermic reaction, necessitating supplemental energy input from sunlight to drive the primary reaction.

Using the following reaction (Equation (1)), the standard free energy change (G°) should be 237.2 kJ mol^−1^.^[^ ^82^, ^83^ ^]^

(1) $$2H_{2}\text{O }\to \;2H_{2}{\;\text{+ O}}_{2}G^{0}\;\text{= }{\;\text{237.2 kJ mol}}^{\text{−1}}$$

Light‐driven water‐splitting reactions over hybrid perovskites involve three intricate processes: 1) charge carrier generation via light absorption, 2) charge carrier separation and transport, and 3) interfacial carrier transfer.^[^ ^84^ ^]^ Three distinct perovskite‐based solar hydrogen production systems have been developed, categorized by their water‐splitting cell structures: photocatalyst systems, photoelectrochemical systems, and PV‐photoelectrochemical systems.

### 5.1 Photocatalysis (Photocatalytic Method) of H_2_

Photocatalysis (PCs) is a chemical process that harnesses light to drive reactions, offering a promising approach for generating hydrogen fuel from water. Upon absorbing sunlight, photocatalysts like titanium dioxide facilitate the splitting of water molecules into hydrogen and oxygen. The resulting hydrogen can be captured and utilized as a fuel source. While this process exhibits potential for high efficiency and cost‐effectiveness, further research is necessary to enhance its performance and reduce costs. Semiconductor materials are commonly employed as photocatalysts to facilitate light‐induced redox reactions, as illustrated in Figure 6a.

**Figure 5:**
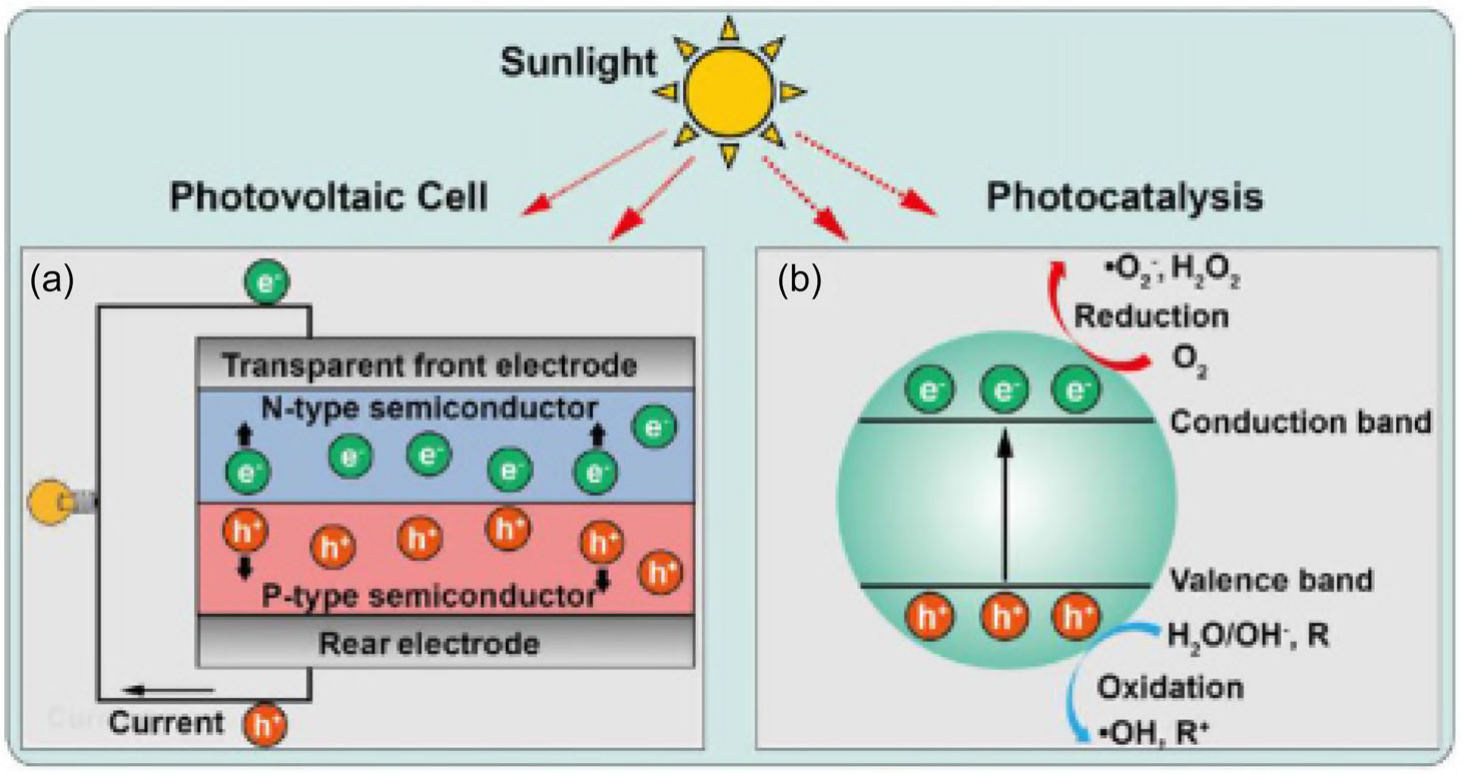
Schematic comparison of a) PV and b) photocatalysis. Reproduced with permission.^[^ ^137^ ^]^

**Figure 6:**
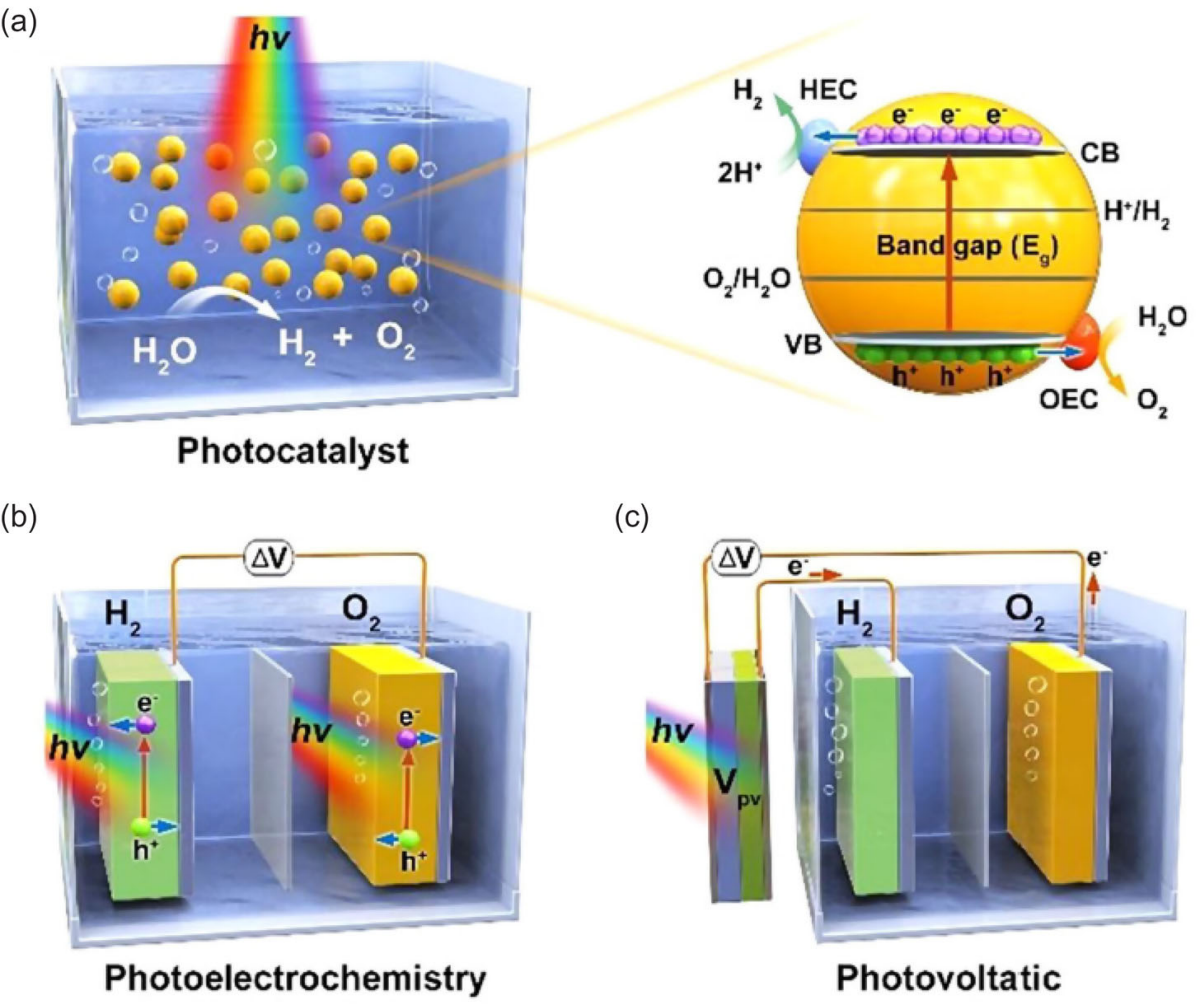
Schematic illustrations of three types of solar water‐splitting system: a) PC system, b) PEC cell, and c) PV‐EC. Reproduced with permission.^[^ ^13^ ^]^

Semiconductor nanoparticles offer tunable optoelectrical properties in photocatalysis systems, influenced by factors such as facet exposure and particle size. The photocatalysis system features a shorter charge transfer channel within particles, rendering it the simplest perovskite‐based water‐splitting device.^[^ ^13^ ^]^ In photocatalytic cells for solar hydrogen production, semiconductor particles are directly injected into the electrolyte (Figure 6a). Upon sunlight illumination, photons with energies exceeding the bandgap (hv ≥ Eg) are absorbed, exciting electrons from the valence band (VB) to the conduction band (CB) and generating energetic electrons and holes (Equation (2), **Figure** **5**b).^[^ ^85^, ^86^ ^]^ These photoexcited charge carriers migrate to the photocatalyst's active surface sites, driving water oxidation (Equation (3)) and proton reduction reactions (Equation (4)) at the semiconductor/electrolyte interface. However, a portion of photoexcited charge carriers undergo recombination at the surface and in the bulk.^[^ ^83^ ^]^

For efficient water splitting, the semiconductor's conduction band edge should be positioned more negatively than 0 V, while the valence band edge should be positioned more positively than 1.56 V, ensuring sufficient potential for photoexcited charge carriers to drive the reaction.^[^ ^87^, ^88^ ^]^ In photocatalytic water splitting, hydrogen and oxygen evolve directly on the surfaces of the same particles, and the resulting gaseous product mixture is collected and subjected to gas separation to yield pure hydrogen.

(2) $$\text{Excitation: hv}\left(\geq E_{g}\right)\to e^{-}+h^{+}$$

(3) $${\text{Oxidation:2H}}_{2}\text{O+4}\;h^{+}\to O_{2}{+\text{4H}}^{+}$$

(4) $${\text{Reduction:2H}}^{+}{+\text{2e}}^{-}\to H_{2}$$

Sacrificial agents, including methanol, ethanol, lactic acid, glycerol, triethanolamine (TEOA), and sodium sulfide/sulfite (Na_2_S/Na_2_SO_3_), act as electron donors. These compounds are readily oxidized by photogenerated holes, thereby reducing electron‐hole recombination rates and redirecting electrons toward proton reduction. This process yields high‐purity molecular hydrogen without concomitant oxygen production].^[^ ^89^ ^]^

### 5.2 Photoelectrochemical System

Photoelectrochemical (PEC) systems utilize light energy to drive electrochemical reactions, facilitating applications such as solar energy conversion, water splitting, and biosensing. A PEC device typically comprises a light‐absorbing material, an electrolyte, and electrodes. Upon illumination, the light‐absorbing material generates excited electrons that drive electrochemical processes in the electrolyte. Efficient PEC operation requires optimal light absorption, charge transport, and current generation. In PEC water‐splitting systems, photocathodes (*p*‐type) and photoanodes (n‐type) work in tandem with an external circuit to facilitate the production of hydrogen and oxygen.

During electrochemical water splitting, the hydrogen evolution reaction (HER) occurs at the cathode, while the oxygen evolution reaction (OER) takes place at the anode. The electric field at the semiconductor‐liquid interface drives minority charge carriers, generated by light absorption, into the solution. The photogenerated electrons and holes are then transferred to molecular catalysts, facilitating the OER and HER reactions. Typically, *p*‐type semiconductors are employed for water reduction, while n‐type semiconductors are used for water oxidation. The mechanism of photoelectrochemical water splitting is illustrated in **Figure** **6**b, with the relevant oxidation and reduction reactions listed below.^[^ ^90^ ^]^

(5) $${\text{2H}}_{2}{\text{O+2e−→H}}_{2}\left(g\right){+\text{2OH}}^{-}{\text{,E}}_{o}\text{=0}\text{.00 V }\left(\text{HER}\right)$$

(6) $${\text{2OH−→1/2 O}}_{2}\left(g\right){+H}_{2}{\text{O+2e}}^{-}\text{, E=1}\text{.23 V }\left(\text{OER}\right)$$

In a PEC cell, the semiconductor‐electrolyte interface facilitates the transfer of photogenerated electrons from the photoanode to the photocathode, enhancing directional charge carrier flow. An external bias can be applied to the circuit to further optimize efficiency. Hybrid perovskite films can be fabricated with or without modifications and utilized as photoelectrodes.

### 5.3 Photovoltaic‐Photoelectrochemical System

A photovoltaic‐photoelectrochemical (PV‐PEC) system integrates PV and photoelectrochemical principles to enhance energy conversion efficiency. The system comprises a PV panel, a photoelectrochemical cell, and a power supply unit. The PV panel generates electrical current from sunlight, which powers the photoelectrochemical cell, driving chemical reactions that produce hydrogen and oxygen. PV‐PEC systems offer advantages such as higher efficiencies, longer lifetimes, and lower costs compared to traditional solar cells. However, system complexity and specialized component requirements increase costs and installation challenges. Notably, hybrid perovskite films can be used in a photovoltaic‐electrocatalytic (PV‐EC) configuration, where the films are placed outside the solution and connected to electrocatalysts immersed in it, as illustrated in Figure 6c.

Hybrid perovskite films can be integrated with other semiconducting components to form photoelectrodes, where the perovskite acts as a PV cell, providing an additional photoinduced potential to drive reactions on the photoelectrode surface. This configuration can be extended to form a tandem PV‐photoelectrocatalytic device. Alternatively, the hybrid perovskite film can be attached to a photoelectrode and placed outside the electrolyte cell, forming an external PV‐PEC tandem architecture (Figure 6c). In this setup, electrocatalysts replace photoelectrodes, and the PV cell is the sole light absorber, converting incident solar energy. To initiate stand‐alone water splitting, the electrolyzer requires an open‐circuit voltage from the PV cell, typically exceeding 1.6 V.^[^ ^13^ ^]^

The choice of cell configuration for hybrid perovskite materials depends on their properties and preparation complexity, including light absorption spectrum, carrier diffusion length, film formation, and encapsulation techniques. PV cells are suitable for materials with short carrier diffusion lengths or those that are challenging to integrate with conducting substrates.^[^ ^13^ ^]^ In contrast, photoelectrochemical cell designs are preferred for hybrid perovskites that can be easily deposited as films and efficiently encapsulated. Both PV‐EC and PEC designs can utilize PV‐class materials with suitable encapsulations. Tandem PC and PEC or PV‐EC configurations are advantageous when the hybrid perovskite exhibits complementary light absorption properties. For large‐scale hydrogen production, PV‐EC systems are the most practical application of hybrid perovskites, requiring high Voc, power conversion efficiency, and efficient electrocatalysts for water oxidation and reduction reactions.^[^ ^13^ ^]^

## 6 Application Methods for Perovskite Materials in Solar Water Splitting

**Figure 7:**
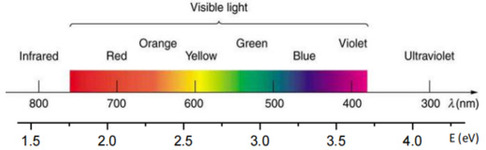
The bandgap value of semiconductors (eV) and the onset wavelength of UV‐vis absorption.

Developing high‐performance photocatalysts requires consideration of three key processes: generation, migration, and reaction of photogenerated charge carriers.^[^ ^20^ ^]^ Perovskites, with their unique properties, are an attractive class of photocatalysts for solar water splitting. Various strategies have been explored to optimize the three crucial processes in perovskite‐based photocatalytic water splitting: photoexcited electron‐hole pair generation, electron‐hole pair separation, and charge carrier migration.^[^ ^20^ ^]^ Notably, perovskite‐based photocatalysts can operate across a broad spectral range, including UV, visible, and IR regions, as illustrated in **Figure** **7**.

Significant research efforts have focused on stabilizing hybrid perovskites under operando photocatalytic reaction conditions and enhancing their photocatalytic activity, yielding notable progress and various stabilization/activity enhancement strategies. However, a comprehensive analysis and systematic summary of these advances are lacking. This section reviews recent developments in methods to improve the stability and photocatalytic activity of hybrid perovskites, focusing on four key strategies.

### 6.1 Enhancements to Chemical Components (Elemental Doping)

The chemical composition of hybrid perovskite materials can be tailored to modulate their optical, electrical, and physical properties. Specifically, variation of the halide content enables adjustment of the bandgap, thereby influencing absorption and emission characteristics. Furthermore, modification of the organic component can enhance thermal stability and resistance to moisture, among other physical properties. By judiciously modifying the chemical structure, hybrid perovskite materials can be optimized for enhanced performance in photocatalytic applications.

Modifying the A, B, and O sites in perovskite structures via doping can tune the bandgap, thereby enhancing light absorption in the visible and near‐infrared (NIR) regions.^[^ ^41^ ^]^ Transition metal doping introduces donor defects, improving electron mobility due to the high atomic energy levels of transition metals without perturbing the conduction band minimum of the host material.^[^ ^91^ ^]^ This modification alters the bandgap, increasing the photocatalytic efficacy of the material. Furthermore, doping creates new transition energy levels, extending absorption to longer wavelengths while preserving the intrinsic absorption properties. By incorporating dopants, the electronic band structure of perovskite photocatalysts can be engineered for optimized performance.^[^ ^38^ ^]^

The valence band and conduction band of hybrid perovskites are primarily composed of d0 transition metal orbitals and 2p atomic orbitals of oxygen or halogen, respectively. Introduction of new elements with p or d orbitals can modify the VB and CB positions via energy level hybridization. Strategic elemental doping can also create mid‐gap states within the bandgap, altering the material's electronic structure.^[^ ^13^ ^]^ Perovskite oxides based on Ti, Ta, and Nb are prominent materials for solar water splitting in the UV and visible regions due to their tunable electronic properties.^[^ ^92^ ^]^ For example, SrTiO_3_ perovskite exhibits a wide bandgap (3.2 eV), limiting its visible light photocatalytic activity. However, doping with transition metals such as Fe, Rh, Ru, Ir, and Mn can reduce the bandgap, enabling visible light‐driven photocatalysis.^[^ ^93^, ^94^, ^95^ ^]^ Notably, NaTaO_3_ photocatalysts have demonstrated high water‐splitting activity, achieving an H_2_ generation rate of 1280 μmol g^−1^ h^−1^ without cocatalysts.^[^ ^96^, ^97^ ^]^ Additionally, atomic defects can alter the crystal structure, impacting the photocatalyst's performance.

Xu et al.'s recent study on LaTa_2_O_6.77_N_0.15_ photocatalysts with a double perovskite structure, synthesized via exfoliation of RbLaTa_2_O_6.77_N_0.15_, demonstrated enhanced 2D charge transport activity. The removal of Rb atoms yielded a material with improved intrinsic oxide perovskite properties, achieving a hydrogen production rate of 160 μmol g^−1^ h^−1^.^[^ ^98^ ^]^ Dopant atoms often occupy interstitial lattice sites without substituting lattice atoms. The incorporation of specific dopants can enhance photocatalytic activity by reducing the bandgap and increasing oxygen vacancies, thereby improving light absorption and reactant adsorption/activation.^[^ ^13^ ^]^

### 6.2 Bandgap Engineering

The bandgap, defined as the energy difference between the valence band maximum and conduction band minimum, plays a crucial role in determining the optoelectronic properties of perovskite materials. **Table** **3** demonstrates the tunability of the bandgap in methylammonium lead halide perovskites (MAPb(I_1–*x*_Br_*x*_)_3_) by adjusting the iodide‐to‐bromide ratio. As the bromide content increases (*x* → 1), the bandgap widens systematically from 1.56 eV (MAPbI_3_) to 2.23 eV (MAPb(Br_0.95_I_0.05_)_3_).^[^ ^99^ ^]^ This bandgap tunability is vital for optimizing the absorption spectrum in solar cells, particularly for designing high‐efficiency tandem PV devices. Halide substitution enables bandgap engineering, allowing for tailored perovskite materials suited for specific layers and applications in solar cell architectures.

**Table 3:** Bandgap tunability in mixed‐halide perovskites.

Devices with perovskite materials	Bandgap [eV]
MAPbl_3_	1.56
MAPb(I_0.88_Br_0.12_)_3_	1.62
MAPb(I_0.74_Br_0.26_)_3_	1.69
MAPb(I_0.58_Br_0.42_)_3_	1.79
MAPb(I_0.41_Br_0.59_)_3_	1.96
MAPb(I_0.28_Br_0.72_)_3_	2.01
MAPb(I_0.05_Br_0.95_)_3_	2.23

Bandgap engineering of hybrid perovskite materials enables precise control over their optoelectronic properties, tailoring them for specific applications. This technique involves modifying the bandgap through chemical alterations, such as halide anion substitution or organic molecule incorporation. By adjusting the bandgap, the optical and electrical properties of the material can be optimized, enhancing the efficiency of optoelectronic devices like solar cells and LEDs. Furthermore, bandgap engineering allows for the creation of materials with tailored spectral responses, thereby optimizing device performance.

Bandgap engineering of hybrid perovskite materials for water splitting aims to optimize their properties for efficient solar energy conversion to hydrogen fuel. By tailoring the bandgap through doping, compositional tuning, or heterojunction formation, the material's performance in water splitting can be significantly enhanced. This technique enables precise control over light absorption properties, allowing for optimized absorption in specific wavelength ranges. Hybrid perovskite materials, with their high charge transport efficiency, can be combined with other materials to further boost the overall efficiency of the water‐splitting process. As a promising research area, bandgap engineering of hybrid perovskite materials holds the potential for revolutionizing solar water splitting efficiency.

**Figure 8:**
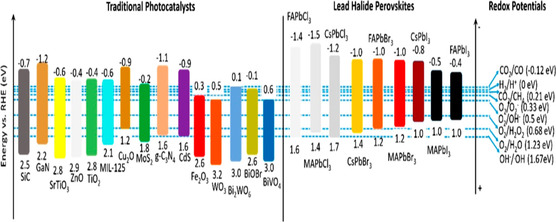
The relative positions of band alignments for different MHPs, traditional photocatalysts, and redox potentials of common half‐reactions in relation to the reversible hydrogen electrode (RHE). Reproduced with permission.^[^ ^100^ ^]^

**Figure** **8** illustrates the band alignment of various metal halide perovskites (MHPs) relative to conventional photocatalysts and common redox half‐reactions. This comparison reveals the thermodynamic compatibility of MHPs for facilitating reduction reactions, such as hydrogen evolution and CO_2_ reduction. Additionally, MHPs with wider bandgaps, particularly chloride‐based materials, exhibit the potential for oxidation reactions.^[^ ^100^ ^]^

Some perovskite materials, due to their large bandgap, respond primarily to UV light, which constitutes a small fraction of solar energy, rather than visible light. The conduction band of perovskites typically features a d‐orbital with an energy level near 0 eV, while the valence band, composed of 2p orbitals, often has an energy level above 3 eV.^[^ ^41^ ^]^ By carefully selecting anions and cations for the A‐site, B‐site, and X‐site during ABX_3_ synthesis, the bandgap of oxide perovskites can be precisely controlled. According to Zhou et al. the energy band structure of A_5_B_4_O_15_ materials can be modified by adjusting the elements or concentrations of A‐ and B‐site ions.^[^ ^101^ ^]^ The perovskite bandgap provides sufficient redox potential for various photocatalytic reactions, including H_2_ production, CO_2_ reduction, and pollutant degradation. Furthermore, incorporating donor and acceptor impurities into the perovskite crystal structure enables bandgap tuning, allowing for optimized photocatalytic performance.^[^ ^41^ ^]^

According to Oshima et al. the conduction‐band potential of HCa_2‐x_Sr_x_Nb_3_O_10_ nanosheets shifts favorably with increasing Sr concentration.^[^ ^102^ ^]^ Huang's team demonstrated a CsPbBr_3–*x*_I_*x*_ bandgap funnel structure, where the bandgap is smallest at the surface (iodide‐rich composition) and increases in the interior (bromide‐rich regions). A similar trend was observed in the MAPbBr_3−*x*_I_*x*_ semiconductor.^[^ ^103^ ^]^

Bandgap tuning methods, including electron‐hole pair recombination suppression and defect engineering, play a crucial role in optimizing photocatalyst performance. The migration of photoinduced charge carriers to the surface triggers redox reactions, while recombination can occur in the bulk or at the surface. Effective suppression of electron‐hole pair recombination is essential for developing efficient solar water‐splitting devices based on hybrid perovskite materials.^[^ ^104^, ^105^, ^106^ ^]^

The development of efficient solar water‐splitting devices relies heavily on suppressing photogenerated electron‐hole pair recombination in hybrid perovskite materials. Techniques such as passivation with dielectric layers, bandgap optimization, and controlled defect introduction can achieve this. Passivation involves depositing a thin dielectric layer (e.g., Al_2_O_3_, HfO_2_, or TiO_2_) on the perovskite surface, acting as an insulator to prevent electron‐hole recombination. Bandgap optimization and controlled defect introduction can also reduce recombination rates. These techniques have been shown to significantly enhance water‐splitting performance. Additionally, the material's symmetry and diffusion length impact charge migration. The diffusion length, which determines the distance photogenerated charges travel before recombining, is critical in the absence of a potential gradient. Materials with longer diffusion lengths enable charge separation beyond the typical charge separation range.

### 6.3 Heterojunction

A promising approach for water splitting involves the use of heterojunctions based on hybrid perovskite materials. These heterojunctions comprise two or more distinct materials integrated into a single unit, enabling efficient water splitting.

Heterojunctions typically combine materials with different bandgap energies to enhance charge separation, thereby increasing the overall efficiency of the water‐splitting process. Additionally, heterojunctions based on hybrid perovskite materials can improve stability by forming a more robust structure through interactions between the constituent materials. This enhanced stability enables the material to withstand extreme conditions, such as high temperatures and intense light, making it suitable for water‐splitting applications.

Heterojunctions can be formed by combining p‐type and n‐type semiconductor materials. Upon contact, free electrons migrate from the n‐type to the p‐type material, creating interfaces with opposing charges and enhancing the electric field within the p‐n junction. This built‐in electric field facilitates the separation and migration of photogenerated charge carriers under light illumination, thereby improving photocatalytic performance (Figure 7).^[^ ^107^, ^108^ ^]^ Examples of semiconductor‐semiconductor photocatalysts include SnO_2_‐TiO_2_, CdS‐TiO_2_, Bi_2_O_3_‐Bi_2_WO_6_, and Bi_2_WO_6_‐TiO_2_.^[^ ^109^ ^]^

The inherent tradeoff between redox capability and sunlight absorption necessitates a compromise, as larger bandgaps enhance redox potential, whereas narrower bandgaps facilitate broader sunlight absorption. A viable solution involves creating heterojunctions that combine two semiconductors with complementary properties, thereby optimizing both redox capability and solar absorption.^[^ ^110^ ^]^ Recent studies have explored various perovskite‐based heterojunction structures for solar water splitting. Notably, Nashim et al. synthesized La_2_Ti_2_O_7_/LaCrO_3_ heterojunctions via a solid‐state approach, demonstrating significantly enhanced solar‐driven water‐splitting performance under visible light irradiation. This improvement is primarily attributed to the synergistic effects of visible‐light activation of La_2_Ti_2_O_7_ and reduced charge carrier recombination facilitated by the heterojunctions.^[^ ^111^, ^112^ ^]^

Researchers have also investigated solid solutions comprising distinct perovskite components to overcome the limitations imposed by wide energy bandgaps in solar water splitting. Examples include Na_0.5_La_0.5_TiO_3_‐LaCrO_3_, AgNbO_3_‐SrTiO_3_, SrTiO_3_‐LaTiO_2_N, and BiFeO_3_‐SrTiO_3_ (BFO‐STO).^[^ ^111^, ^113^, ^114^, ^115^, ^116^ ^]^ The band structure of these materials can be effectively tuned by introducing nonmetal elements (e.g., C, N, S), 3d transition elements, or cations with d^0^, d^10^, or d^10^s^2^ electronic configurations.^[^ ^111^, ^117^, ^118^, ^119^, ^120^ ^]^

The separation of electron‐hole pairs can be enhanced through optimal heterojunction contact between two semiconductors, which induces band bending and extends the band bending region.^[^ ^121^ ^]^ Heterojunctions are categorized into three types based on the band alignment of the constituent semiconductors. A detailed explanation of each heterojunction type can be found in refs. [13, 20]. Notably, type II heterojunctions are typically preferred for composites comprising two n‐type semiconductors.

Furthermore, Z‐scheme heterojunctions can enhance charge separation while maintaining the high redox potential of semiconductors. This concept has been demonstrated in a well‐designed α‐Fe_2_O_3_/amine‐RGO/CsPbBr_3_ system, as reported by Kuang et al. The matching energy band structure facilitated directional charge transfer from α‐Fe_2_O_3_ to CsPbBr_3_, effectively suppressing photogenerated carrier recombination.^[^ ^122^ ^]^ Additionally, certain ferroelectric perovskite materials exhibit outstanding electrical polarization properties, which can create a ferroelectric‐induced charge separation field surrounding the particles.^[^ ^13^ ^]^

**Figure 9:**
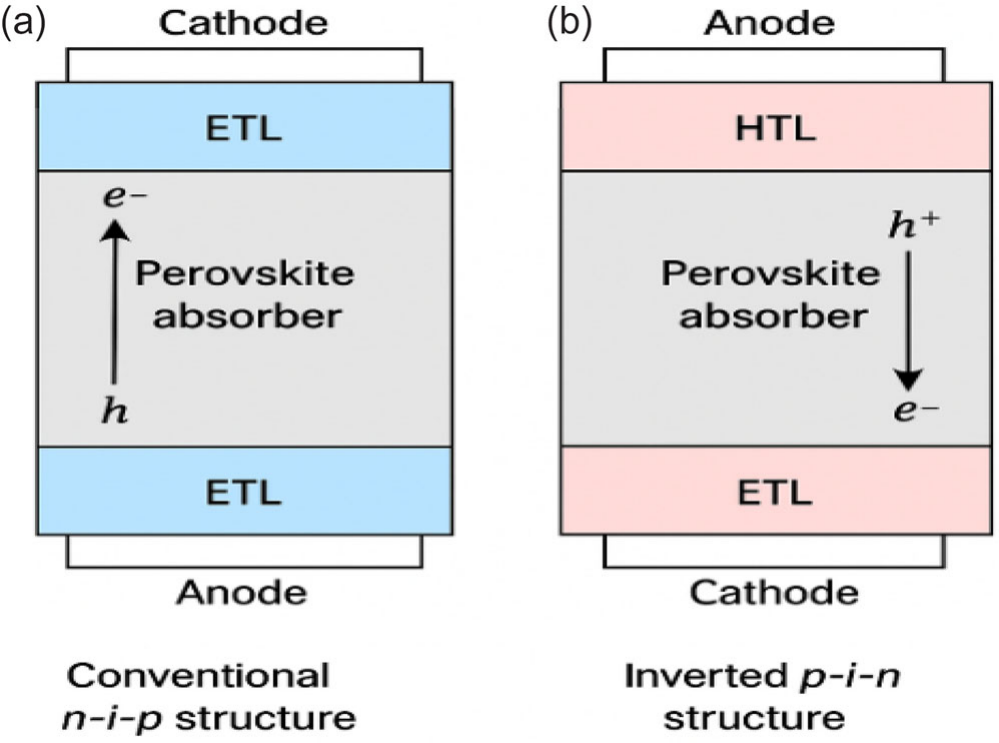
Schematic illustration of heterojunction architectures in perovskite solar cells. a) Conventional n–i–p structure with electron transport layer (ETL), perovskite absorber, and hole transport layer (HTL). Transparent conductive oxide (TCO). b) Inverted p–i–n structure demonstrating reversed carrier flow.

The construction of heterojunction interfaces is crucial for perovskite solar cells, as it enhances both charge separation and transportation efficiency, directly impacting performance metrics. A schematic illustration of typical heterojunction configurations, including n‐i‐p and p‐i‐n structures in PSCs, is provided in **Figure** **9**.^[^ ^123^ ^]^

### 6.4 Ferroelectric Property Application

Ferroelectric materials exhibit spontaneous electric polarization, which can be reversed under an applied electric field and retained upon its removal. Specific perovskite materials displaying ferroelectricity have been utilized in various applications, including capacitors, nonvolatile memory storage, actuators, and piezoelectric devices that facilitate the conversion of mechanical energy into electrical energy. Additionally, ferroelectric materials can be employed as electrodes for water splitting, leveraging their ability to store and release electrical charge to facilitate redox reactions. Notably, these materials can serve as catalysts for both hydrogen evolution reaction (HER) and oxygen evolution reaction (OER), crucial steps in the water‐splitting process. Furthermore, ferroelectric materials can enhance the efficiency and cost‐effectiveness of capacitive deionization (CDI) systems for water treatment, reducing energy consumption and increasing system lifetime. Ferroelectric materials offer a promising alternative to conventional semiconductors in solar water splitting, leveraging their exceptional ability to efficiently separate and transport photoexcited charge carriers.^[^ ^90^ ^]^ Notable perovskite ferroelectric materials, such as BiFeO_3_ (BFO) and Pb(Zr,Ti)O_3_ (PZT),^[^ ^124^, ^125^, ^126^, ^127^ ^]^ exhibit stable and adjustable residual ferroelectric polarization. This polarization generates a uniform electric field, facilitating charge separation and controlled charge transfer. In composite materials, the intrinsic ferroelectric field can enhance charge separation, improving overall performance.

Systematic photoelectrochemical investigations have demonstrated that the direction of charge transfer of photoexcited charges in bismuth ferrite and surface modifiers, such as molecular dyes and CdSe quantum dots (QDs), can be controlled by adjusting the poling conditions of ferroelectric photoelectrodes. This finding highlights the potential of tunable polarization fields in ferroelectric perovskite materials to facilitate PEC reactions, enabling novel PEC system designs for simultaneous water oxidation and reduction on a single photoelectrode.^[^ ^128^ ^]^ Further research, such as Song et al.'s comprehensive summary on the impact of ferroelectric switching on PEC behavior under various polarization states,^[^ ^129^ ^]^ exemplifies the exploitation of ferroelectric perovskite materials for PEC water splitting.

## 7 Summary of Firms/Companies Commercializing Perovskites

The rapid advancement of perovskite solar cells has prompted companies to focus on module manufacturing, primarily targeting large‐area devices (> 100 cm^2^). Techniques such as roll‐to‐roll printing,^[^ ^130^ ^]^ inkjet printing,^[^ ^131^ ^]^ and screen printing^[^ ^132^ ^]^ are employed to fabricate perovskite films. Companies like Toshiba,^[^ ^133^ ^]^ Saule Technologies,^[^ ^131^ ^]^ and Energy Materials Corporation are developing flexible perovskite solar modules.^[^ ^130^ ^]^ Oxford PV is set to produce PVK/c‐Si tandem cells following the establishment of a dedicated production line.^[^ ^134^ ^]^ For a comprehensive overview of company developments and progress, readers are referred to references.^[^ ^39^, ^100^, ^135^, ^136^ ^]^

## 8 Summary and Perspectives

As the world intensifies its pursuit of sustainable and affordable clean energy, the persistent energy crisis driven by increasing energy demands and reliance on coal‐based carbonization remains a pressing concern. This study explores a meaningful shift toward mitigating the environmental impacts of perovskite PV devices and energy generation via water splitting. The motivation stems from the urgent need to reduce ecological harm during both the manufacturing and end‐of‐life phases of perovskite solar cells. Advancements in this field are crucial for positioning hydrogen production through perovskite‐based systems as a viable and cleaner alternative to conventional fossil fuels.

The abundance of solar resources on our planet renders them a vital component for renewable energy generation. This review provides an overview of hybrid perovskite‐based photocatalysts, with a focus on their development for solar‐driven hydrogen production. Photocatalysis offers a promising approach to harnessing solar energy to address pressing environmental and energy challenges. Elucidating the fundamental mechanisms underlying photocatalysis is essential for designing highly efficient photocatalysts. This article highlights the operational principles and critical properties of perovskite materials, underscoring their pivotal role in photocatalytic processes.

This review discusses three solar hydrogen production systems: photocatalytic, photoelectrochemical, and PV‐electrochemical systems. Strategies to enhance cell performance include elemental doping, heterojunction formation, bandgap engineering, and exploitation of ferroelectric properties. Particular emphasis is placed on methods to improve the stability of hybrid perovskite‐based semiconductors. Despite significant advances in hybrid perovskite device efficiency for solar water splitting, opportunities remain for innovative research on advanced devices. Future directions include exploring novel hybrid perovskite materials, designing innovative device structures, and experimentally evaluating large‐scale hydrogen production systems. Given the unique optoelectronic properties of hybrid perovskites, further expansion of their applications in photocatalysis is anticipated.

## Conflict of Interest

The authors declare no conflict of interest.
